# Cellular imaging of endosome entrapped small gold nanoparticles

**DOI:** 10.1016/j.mex.2015.06.001

**Published:** 2015-06-10

**Authors:** Chang Soo Kim, Xiaoning Li, Ying Jiang, Bo Yan, Gulen Y. Tonga, Moumita Ray, David J. Solfiell, Vincent M. Rotello

**Affiliations:** Department of Chemistry, University of Massachusetts Amherst, 710 North Pleasant Street, Amherst, MA 01003, USA

**Keywords:** Confocal measurement of cellular uptake of gold nanoparticles, Gold nanoparticles, Confocal laser scanning microscopy, Nanoparticle quantification, ICP-MS, Reflectance imaging

## Abstract

Small gold nanoparticles (sAuNPs, <10 nm in a core diameter) have been used for drug delivery and cancer therapy due to their high payload to carrier ratio. Information about the amount and location of sAuNPs in cells and tissues is critical to many applications. However, the current detection method (i.e., transmission electron microscopy) for such sAuNPs is limited due to the extensive sample preparation and the limited field of view. Here we use confocal laser scanning microscopy to provide endosome-entrapped sAuNP distributions and to quantify particle uptake into cells. The quantitative capabilities of the system were confirmed by inductively coupled plasma-mass spectrometry, with an observed linear relation between scattering intensity and the initial cellular uptake of sAuNPs using 4 nm and 6 nm core particles.

The summary of the method is:

• This non-invasive imaging strategy provides a tool for label-free real-time tracking and quantification of sAuNPs using a commercially available confocal laser scanning microscope.
• Scattering intensity depends on particle size.
• The linear relation established between scattering intensity and uptaken gold amount enables simultaneous quantitative assessment through simple image analysis.

## Graphical abstract

**Figure:**
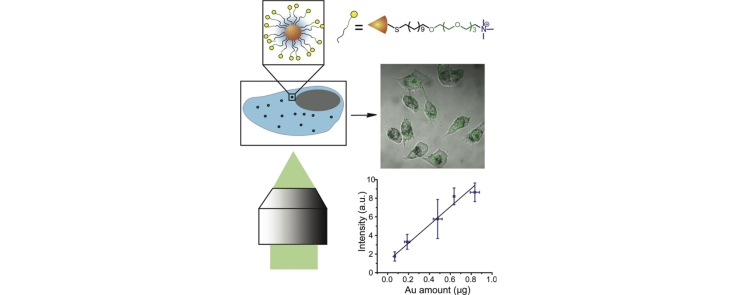

## Method details

We report a simple, rapid, and non-invasive approach for the imaging of sAuNPs within cells by using a standard confocal laser scanning microscope (CLSM). No additional optical or imaging system is required for this approach. A single-wavelength laser excitation was used to excite sAuNPs within the cell, and the reflective images were recorded to explore the size-dependent visibility of the AuNPs. These studies demonstrate that sAuNPs as small as 4 nm in core size can be readily imaged. Image analysis was carried out to explore the correlation between the sAuNP scattering intensity and sAuNP quantities inside cells.

### Step 1: surface-functionalized gold nanoparticle synthesis

#### Materials

All the reagents/materials required for nanoparticle synthesis were purchased from Fisher Scientific, except for hydrogen tetrachloroaurate(III) hydrate, which was obtained from Strem Chemicals Inc. The organic solvents were from Pharmco-Aaper or Fisher Scientific and used as-received except for dichloromethane, which was distilled in the presence of calcium hydride. HeLa cells (human cervical-cancer cell line) were purchased from ATCC. Dulbecco’s Modified Eagle’s Medium (DMEM; Sigma, D5523) and fetal bovine serum (FBS; Fisher Scientific, SH3007103) were used in cell culture.

#### Procedure

Gold nanoparticles (AuNPs) were synthesized and characterized according to previous reports with slight modifications [1]. Briefly, Brust–Schiffrin two-phase synthesis method [2] was used to prepare dodecanethiol-protected AuNPs (AuNPs-DT) with 2 nm core diameter. AuNPs-DT (4 and 6 nm) were grown from 2-nm AuNPs according to Miyake’s heat-induced size-evolution strategy [3] with a slight modification.

- 2-nm AuNPs-DT were heated to 154 °C and 165 °C for 4 and 6 nm AuNPs-DT, respectively, with a heating rate of 2 °C/min and held for 30 min at that temperature.
- Murray’s place-exchange method [4] was then used to prepare functionalized AuNPs.

The sizes of AuNPs were characterized by TEM and dynamic light scattering (DLS) (Fig. S2). The surface functionalities of AuNPs have been characterized by proton nuclear magnetic resonance (^1^H NMR) and laser desorption/ionization mass spectroscopy (LDI-MS) (Figs. S3 and S4). Zeta-potential values were measured using a Malvern Zetasizer Nano ZS instrument.

### Step 2: cell culture and cellular uptake of AuNPs

Prior to the cellular uptake study of AuNPs, HeLa and MCF 7 cells were seeded into a 24-well plate at a density of 25,000 cells/well with low-glucose DMEM supplemented with 10% FBS and 1% antibiotic.

#### Procedure

- The cultures were maintained at 37 °C in a humidified atmosphere with 5% CO_2_.
- After 24 h of seeding, the cells were washed once with PBS and exposed to DMEM solution containing either 4- or 6-nm AuNPs at different concentrations (2.5, 10, 20, 40, and 60 nM for 4-nm and 0.7, 2.7, 5.5, 10.9, and 16.4 nM for 6-nm).
- Replicated wells containing the cells and cell-culture medium only (no AuNPs) were prepared as the negative control.
- After 3 h incubation, the cells were washed three times with PBS to remove extra nanoparticles and used for imaging as well as ICP-MS quantification.

### Step 3: ICP-MS sample preparation

#### ICP-MS instrumentation

All ICP-MS measurements were performed on a PerkinElmer Elan 6100. Operating conditions of the ICP-MS were: rf power, 1550 W; plasma Ar flow rate, 15 L/min; nebulizer Ar flow rate, 0.96 L/min; isotopes monitored, ^197^Au; dwell time, 50 ms; nebulizer, cross-flow; spray chamber, Scott.

#### Procedure

After cellular uptake of the AuNPs, the lysed cells were digested with 0.5 mL fresh aqua regia (highly corrosive!) for 10 min. The digested samples then were diluted to 10 mL with deionized water. A series of gold standard solutions (20, 10, 5, 2, 1, 0.5, 0.2, and 0 ppb) were prepared before each experiment. Each gold standard solution also contained 5% aqua regia. Each standard solution was measured five times by ICP-MS using the operating conditions described above. The resulting calibration curve was used to determine the gold amount taken up by the cells in each sample.

### Step 4: imaging by confocal laser scanning microscopy

Imaging was carried out with an inverted confocal laser scanning microscopy in reflection mode, Zeiss 510 (Carl Zeiss, Jena, Germany) using both 63× and 40× oil immersion objectives.

#### Procedure

- The argon laser at the wavelength of 514 nm (argon, 15 mW) was reflected down to the sample direction by a reflection mirror.
- Dropped the oil on the top of 63× and 40× oil immersion objective lenses.
- Placed the confocal sample on the lenses and uncover the coverslip of the culture dish.
- A dichroic beam splitter (NT 80/20, Carl Zeiss) was used to excite the intracellular AuNPs.
- The laser beam was focused by a lens to form a small spot within 1 mm.
- Light scattering was recorded in a channel with a long pass 505 nm emission filter.
- Images were taken at six different spots of the culture dish.
- The focus of cells was adjusted using the differential inference contrast (DIC) as a reference.

### Step 5: confocal image quantification

ImageJ (version 1.42q, National Institutes of Health) was employed to quantify the weighted means of scattering intensity of AuNPs by selecting a 512 × 512 area. Analyze-histogram was used to create a list of pixel values with corresponding pixel numbers. Background noise level threshold was determined from cell-only images (no AuNPs), and signals that were three times over the threshold were collected and averaged. Five confocal images were analyzed for each set of experiments.

### Methods validation

Cellular imaging of endosome entrapped small gold nanoparticles was validated using a number of measures.

#### Characterization of gold nanoparticles

**Fig. 1:**
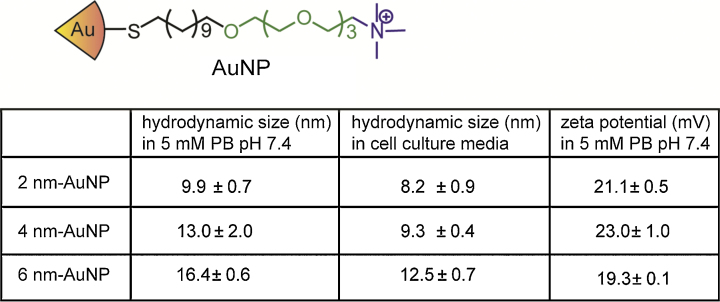
Ligand structure of AuNPs used in this study, and dynamic light scattering analysis and zeta-potential measurements of particles in PB and cell culture media.

Gold nanoparticles with different core sizes (2, 4, and 6 nm in diameter) were chosen to determine the size requirements for optical detection of endosome-entrapped sAuNPs. All sAuNPs were synthesized bearing the same surface functionality (Figs. 1, S1–S3, and Supporting information for detailed synthesis).

#### Cellular uptake and endosome-entrapment of sAuNP

**Fig. 2:**
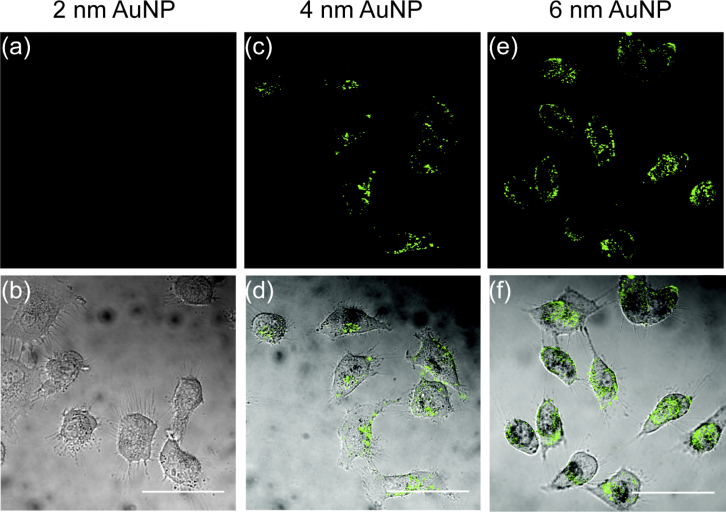
CLSM images of different-sized AuNPs acquired after 3 h incubation in HeLa cells. The scattering images (a, c, and e) and corresponding merged images with bright-field images (b, d, and f) represent incubation with 2-nm (200 nM), 4-nm (100 nM), and 6-nm (30 nM) AuNPs, respectively. Scale bar is 50 μm. (For interpretation of the references to color in text, the reader is referred to the web version of this article.)

HeLa cells were prepared and incubated with the different-sized sAuNPs for 3 h and then washed three times with phosphate-buffered saline (PBS) to remove free sAuNPs from the cell medium before imaging. The images were collected by using CLSM at 514 nm. Cells without AuNPs were used as a control to adjust the detector gain and establish the baseline. As shown in Fig. 2, scattering signals were observed from 6- and 4-nm AuNPs (marked in green) after being dramatically uptaken by the cells. Whereas no signals were detected from 2-nm AuNPs even at the highest concentration of 200 nM. These images reveal that the scattering imaging intensity of sAuNPs is size dependent. Both 4- and 6-nm AuNPs were clearly visualized inside cells due to the high enrichment of the cells with both the particles. Therefore, the 4- and 6-nm AuNPs were used in the following studies. Lysotracker was used and confirmed that the uptaken AuNPs (4 and 6 nm) were partially localized in the late endosome/lysosome at this point (Fig. S3).

#### Quantitative Imaging of endosome-entrapped sAuNP

**Fig. 3:**
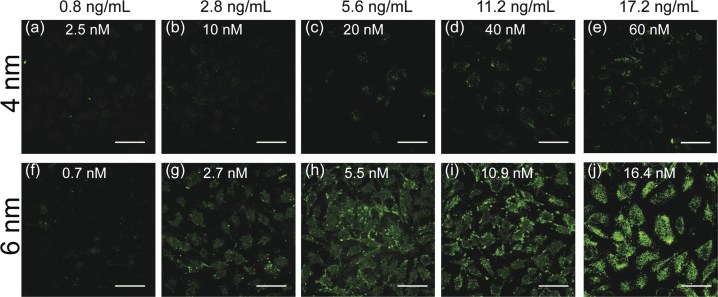
Representative CLSM images of intracellular AuNPs at different concentrations after 3 h of incubation in HeLa cells. (a)–(e) are 4-nm AuNPs and (f)–(j) are 6-nm AuNPs. Scale bars represent 50 μm.

Since the cellular uptake of functionalized AuNPs is dictated by NP concentration [5], we next investigated the concentration effect on the reflective imaging of intracellular AuNPs. The same gold mass concentration was chosen to compare 4- and 6-nm AuNP samples. All images were acquired with the same detector gain to ensure comparable relative intensities. No signals were observed for either NP at the lowest concentrations, and as the concentration increased, the scattering signal intensity increased as well (Fig. 3). At the same incubation concentrations, the 6-nm AuNPs gave rise to stronger signals than the 4-nm AuNPs. For 4-nm AuNPs, visible scattering signals were seen from the concentration of 20 nM (Fig. 3(c)). In the case of 6 nm AuNPs, the concentration of 2.7 nM was high enough to show scattering signals (Fig. 3(g)). These images reveal that the scattering intensity from AuNPs is concentration-dependent. Compared to 4-nm sAuNPs, the cellular uptake of 6-nm sAuNPs is dramatically more and this in turn gives rise to strong scattering signal for 6-nm sAuNPs. This result indicates the potential of our method to provide quantitative information about partial endosome-entrapped sAuNPs in addition to the visualization of intracellular sAuNPs.

**Fig. 4:**
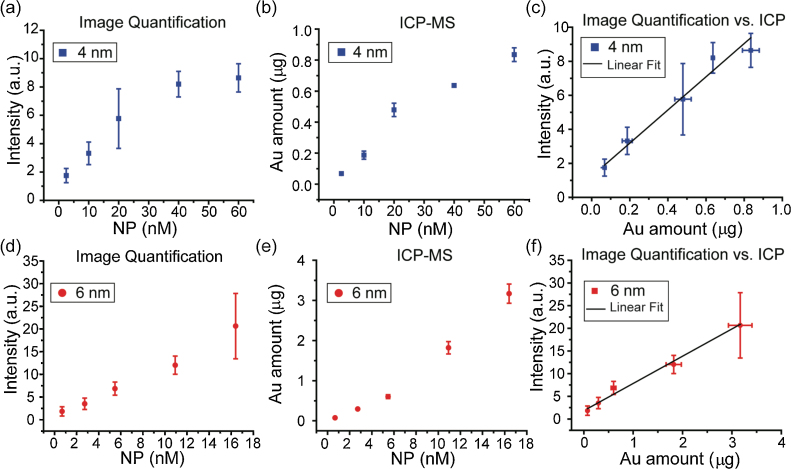
Quantification of the amounts of 4- and 6-nm AuNPs in cells at different concentrations. (a, d) Scattering intensity obtained from CLSM images. (b, e) Au amount measured from ICP-MS. (c, f) Linear fit of calculated reflective intensity and Au amount measured by ICP-MS with corresponding standard deviations.

The correlation between signal intensity obtained from CLSM images and the endosome-entrapped amount of gold was studied by using inductively coupled plasma-mass spectrometry (ICP-MS). HeLa cells were incubated in four replicates with the AuNPs at different concentrations (2.5, 10, 20, 40, and 60 nM for 4-nm and 0.7, 2.7, 5.5, 10.9, and 16.4 nM for 6-nm) for 3 h. The treatment of HeLa cells with different concentrations of AuNP had negligible cytotoxicity to cells, as confirmed by Alamar Blue assay in Fig. S5. After washing, three replicates were lysed for ICP-MS quantification, and one was imaged by CLSM. The microscopy images were analyzed by using ImageJ to assess the weighted mean scattering intensity. The calculated average intensity of 4-nm AuNPs, as shown in Fig. 4(a), increased with increasing AuNP concentration, and the actual cellular uptake of gold amount measured by ICP-MS also increased in a concentration-dependent fashion, as expected (Fig. 4(b)). The average intensity values were then plotted versus endosome-entrapped gold at corresponding concentrations, affording a linear trend *R*^2^ value of 0.97 (Fig. 4(c)). Results from the 6-nm AuNPs displayed a similar trend in both image and ICP-MS quantification, and the average scattering intensity again correlated with endosome-entrapped gold amount in a linear way, with an *R*^2^ value of 0.94 (Fig. 4(f)). The fitting analysis for these two AuNPs indeed demonstrated that our approach provides a rapid quantification method for intracellular sAuNPs. Other than HeLa cells, MCF7 cells were incubated in four replicates with the AuNPs at different concentrations (10, 20, and 40 nM for 4-nm and 2.7, 5.5, and 10.9 nM for 6-nm) for 3 h. Results from the MCF7 cells displayed a similar trend in both image and ICP-MS quantification, and the average scattering intensity again correlated with endosome-entrapped gold amount in a linear fashion (Fig. S6).

### Additional information

#### Background

Small gold nanoparticles (sAuNPs) with a core diameter <10 nm have unique properties linked to their size. For example, the match in the size of these NPs to proteins [6], multi helix DNA bundle structures [7], and nuclear pores [8] is ideal for engineering biomolecule-NP interactions and targeting the cell nucleus; their high biocompatibility is maintained even after they are endocytosed by cells [9]; their large surface-to-volume ratio provides high payload efficiency [10]; and their strong diffusivity enables them to penetrate the blood–brain barrier [11] and solid tumor tissues [12]. These properties make sAuNPs excellent vehicles for various applications including biosensor [13], [14], [15], drug/gene delivery [16], [17], [18], and cancer therapy [19].

With the increasing use of sAuNPs in biomedical applications, especially in diagnosis and therapy [20], it is critical to detect and localize the AuNPs inside cells and to be able to determine their distribution within the cell for a detailed understanding of target sites and potential risks [21], [22]. For intracellular imaging of AuNPs, transmission electron microscopy (TEM) provides high resolution, showing AuNP shapes and sizes in addition to their location [23], [24]. However, identifying sAuNPs requires high magnification, resulting in a limited field of view encompassing a tiny fraction of a single cell [25]. In addition, the extensive sample preparation and fixation required for TEM makes it challenging to explore intracellular trafficking of AuNPs in real time. Optical microscopy, on the other hand, is capable of rapidly screening whole cells and sample preparations are facile. Although AuNPs have been visualized using this approach by exploiting the localized surface plasmon resonance (SPR) [26], [27], [28], [29], [30], [31], detection of sub-10 nm AuNPs remains a challenge because the SPR scattering signal rapidly disappears as size decreases [32], [33]. Photothermal [34] and multispectral imaging systems have been used to visualize small nanoparticles even down to the single particle level [35], however these approaches require specialized equipment that is not widely available.
