# Supplementary material for: Cellular imaging of endosome entrapped small gold nanoparticles
Source: MethodsX. 2015 Jun 10;2:306–15. doi: 10.1016/j.mex.2015.06.001 (PMC4487928; doi:10.1016/j.mex.2015.06.001)
Supplement: Supplementary file 1 [file mmc1.doc]

Supplementary data

Cellular imaging of endosome entrapped small gold nanoparticles

Chang Soo Kim^‡^, Xiaoning Li^‡^, Ying Jiang, Bo Yan, Gulen Y. Tonga, Moumita Ray, David J. Solfiell, and Vincent M. Rotello*

^‡^These authors contributed equally to this work.

Department of Chemistry, University of Massachusetts-Amherst, 710 North Pleasant Street, Amherst, Massachusetts, 01003, USA

*Address correspondence to [rotello@chem.umass.edu](mailto:rotello@chem.umass.edu).


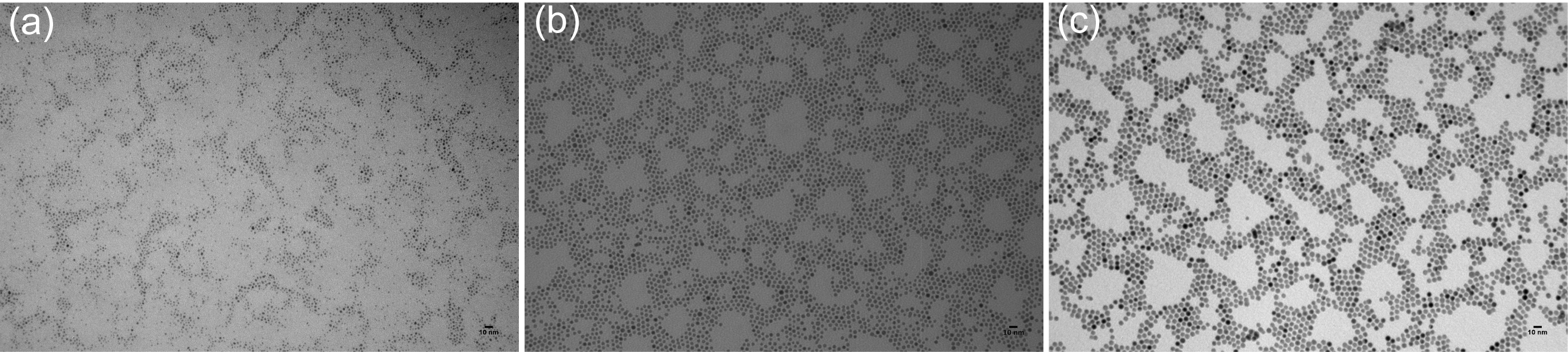


**Fig. S1**. TEM images of different-sized AuNPs. The 2-nm AuNPs (a), 4-nm AuNPs (b), and 6-nm AuNPs (c). Scale bar represents 10 nm.


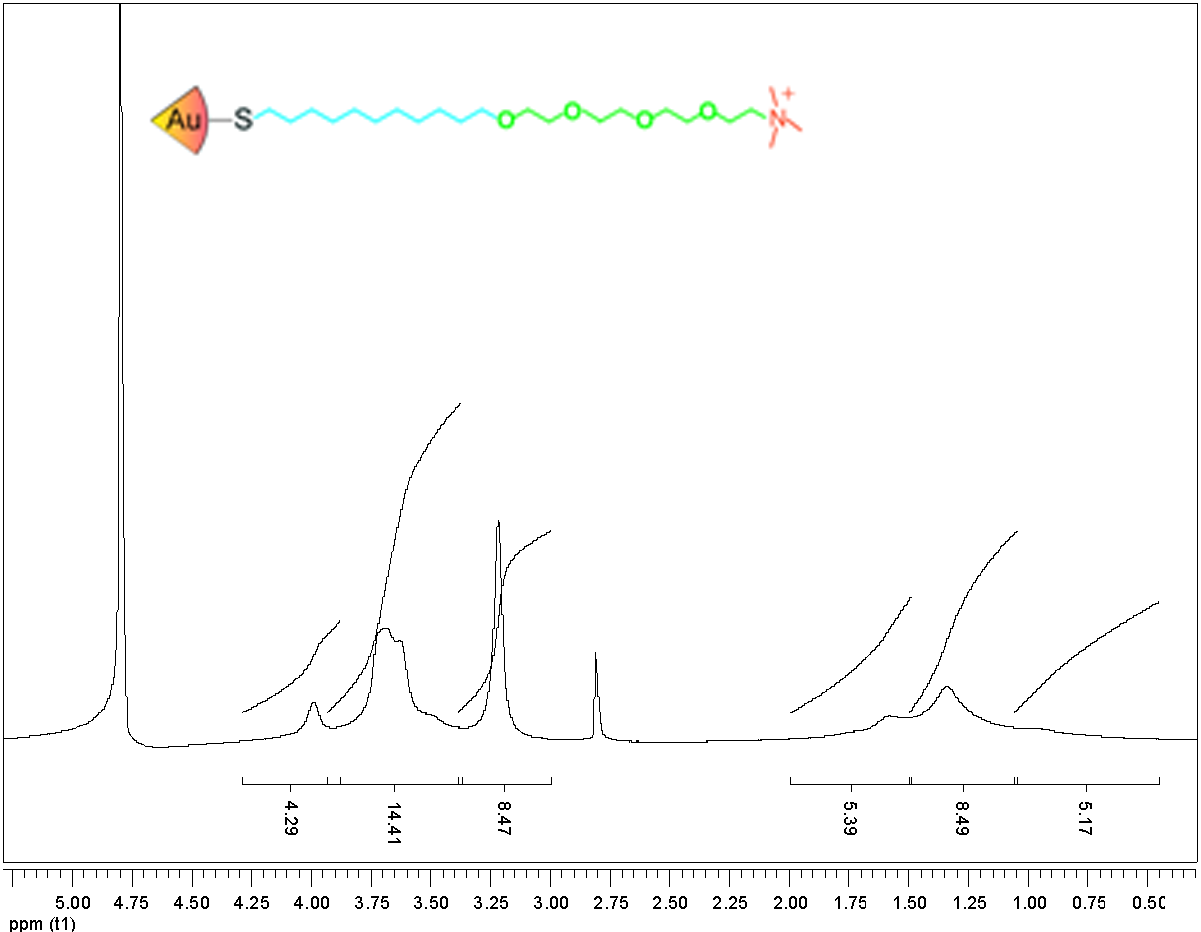


Fig. S2. 400 MHz ^1^H NMR spectra of AuNPs in water-D (D, 99.8%). All AuNPs (2-nm, 4-nm, and 6-nm) have the same ligands.


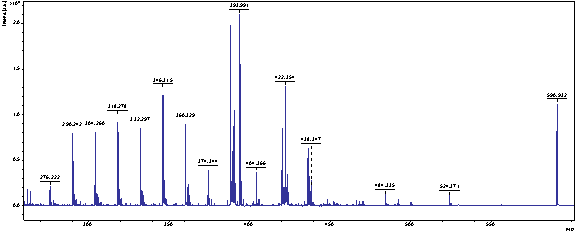


Fig. S3. Laser desorption/ionization mass spectroscopy (LDI-MS) analysis of AuNPs. All the AuNPs (2-nm, 4-nm, and 6-nm) have been characterized by LDI-MS and the surface functionalities have been confirmed following the reported method.

**[[1]](#endnote-2)**


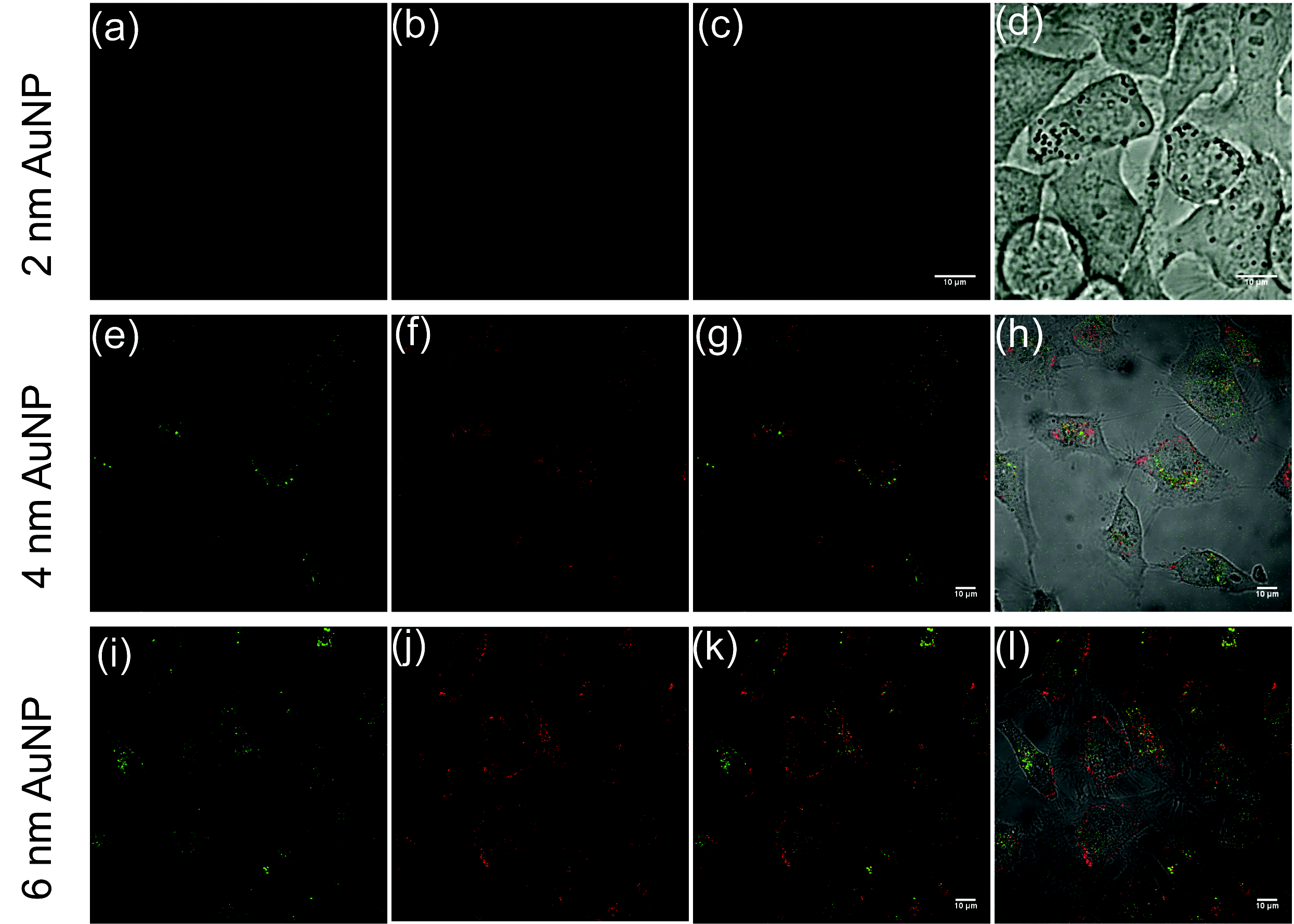


**Fig. S4**. CSLM images of different-sized AuNPs (red) in HeLa cells after using Lysotracker (green). The fluorescent images (a, e, and i), scattering images (b, f, and j), merged images (c, g, and k), and corresponding merged images with bright-field images (d, h, and l). Scale bar represents 10 μm.


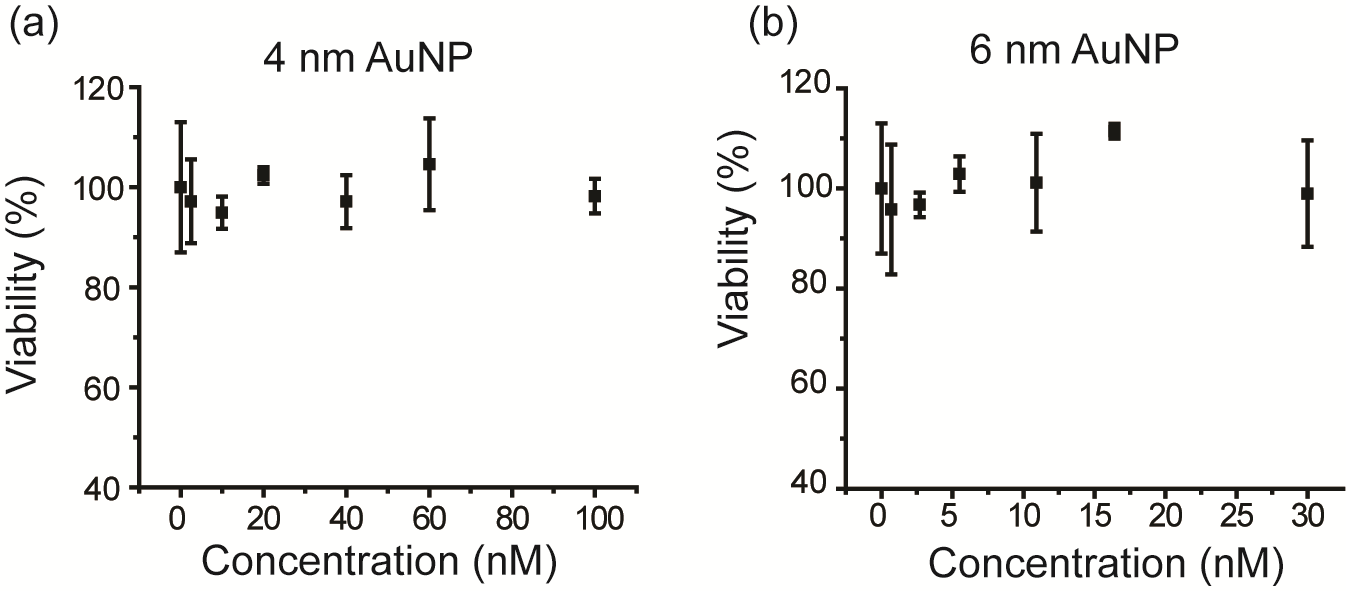


**Fig. S5**. Viability of HeLa cells treated with different concentrations of 4- and 6- nm AuNP in DMEM solution for 3 h.


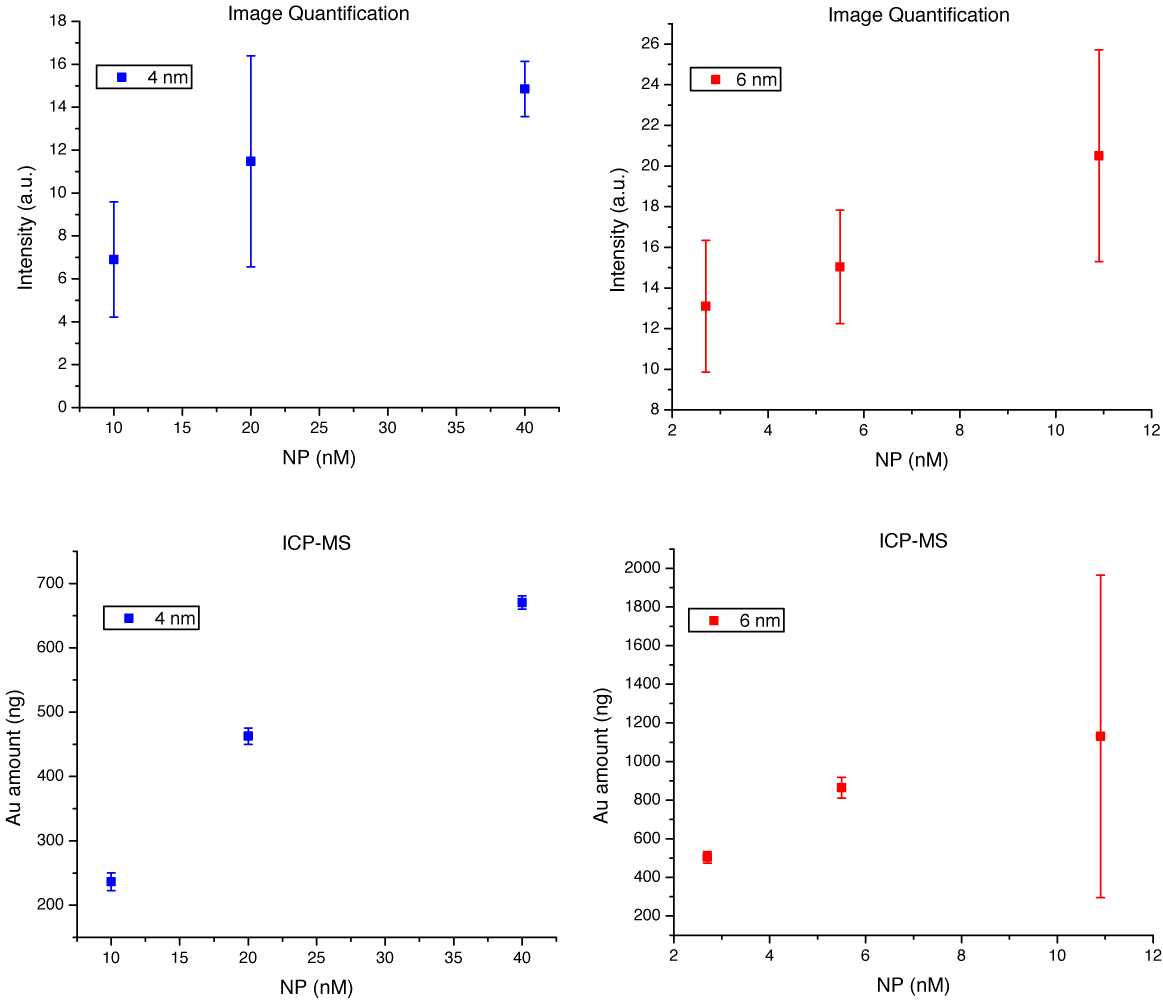


**Fig. S6**. Quantification of the amounts of 4- and 6-nm AuNPs in MCF7 cells at different concentrations.

1. [?] Yan, B.; Zhu, Z.-J.; Miranda, O.R.; Chompooser, A.; Rotello, V.M.; Vachet, R. W., *Anal. Bioanal. Chem*. **2010**, *396*, 1025. [↑](#endnote-ref-2)
